# Rational PCR Reactor Design in Microfluidics

**DOI:** 10.3390/mi14081533

**Published:** 2023-07-31

**Authors:** Masoud Madadelahi, Marc J. Madou

**Affiliations:** 1 School of Engineering and Sciences, Tecnologico de Monterrey, Monterrey 64849, NL, Mexico; masoud.m@tec.mx; 2 Autonomous Medical Devices Incorporated (AMDI), Santa Ana, CA 92704, USA

**Keywords:** polymerase chain reaction, fast PCR, extreme PCR, microfluidics, PCR reactor design, heat transfer, thermal cycling

## Abstract

Limit of detection (LOD), speed, and cost for some of the most important diagnostic tools, i.e., lateral flow assays (LFA), enzyme-linked immunosorbent assays (ELISA), and polymerase chain reaction (PCR), all benefited from both the financial and regulatory support brought about by the pandemic. From those three, PCR has gained the most in overall performance. However, implementing PCR in point of care (POC) settings remains challenging because of its stringent requirements for a low LOD, multiplexing, accuracy, selectivity, robustness, and cost. Moreover, from a clinical point of view, it has become very desirable to attain an overall sample-to-answer time (t) of 10 min or less. Based on those POC requirements, we introduce three parameters to guide the design towards the next generation of PCR reactors: the overall sample-to-answer time (t); lambda (λ), a measure that sets the minimum number of copies required per reactor volume; and gamma (γ), the system’s thermal efficiency. These three parameters control the necessary sample volume, the number of reactors that are feasible (for multiplexing), the type of fluidics, the PCR reactor shape, the thermal conductivity, the diffusivity of the materials used, and the type of heating and cooling systems employed. Then, as an illustration, we carry out a numerical simulation of temperature changes in a PCR device, discuss the leading commercial and RT-qPCR contenders under development, and suggest approaches to achieve the PCR reactor for RT-qPCR of the future.

## 1. Introduction

**Figure 1:**
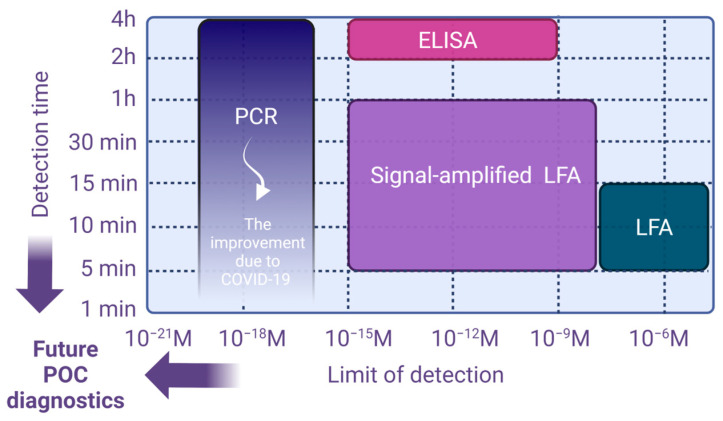
Comparison of the detection limit and detection time for various POC techniques. Improvement in PCR as a “COVID-19 accelerator effect”.

Three of the most essential diagnostic instruments, lateral flow assays (LFA), enzyme-linked immunosorbent assays (ELISA), and polymerase chain reaction (PCR), all benefited from the pandemic’s financial and regulatory support. According to Figure 1, prior to COVID-19, LFA was the quickest and cheapest option, but it had the worst (highest) limit of detection (LOD). PCR was the slowest and most expensive, but had the best (lowest) LOD. ELISA fell between these two extremes in terms of cost, LOD, and efficiency. Today, due to the “COVID-19 accelerator effect”, there are point-of-care (POC) reverse transcriptase quantitative PCR (i.e., RT-qPCR) COVID-19 tests on the market that provide sample-to-result in less than 20 min while maintaining the same extremely low LOD at a reduced cost [1]. Even POC PCR assays that take less than ten minutes are in development, surpassing both LFA and ELISAs in terms of time and LOD [2,3]!

A basic PCR technique consists of three phases that are repeated or cycled: (1) denaturation of the double-stranded DNA target (94–95 °C), (2) annealing depending on the melting temperature of the primer pair (T_m_) between 55 °C and 70 °C (the annealing temperature is typically 5 °C below the T_m_ of the primers, and the optimal annealing temperature for a given primer pair on a particular target is calculated as: T _a opt_ = 0.3 × (T_m_ of primer) + 0.7 × (T_m_ of product) − 14.9 [4]), and (3) extension (72 °C) [5]. For faster analysis, the annealing and extension steps are often mixed after the first starting step. First and foremost, PCR performance is evaluated based on its accuracy and precision; accuracy refers to the veracity of the results, while precision refers to the consistency between repeated tests regardless of their accuracy [6]. Specificity is another crucial characteristic; a PCR method is said to be specific when it generates a single and unique amplification product, that is, the expected target sequence. The efficiency (yield) of a PCR process is a quantitative characteristic; when a PCR method is identified as having a higher efficiency, this means that it can produce a greater number of amplified targets using fewer thermal cycles [7]. The LOD of PCR refers to the lowest level of initial DNA target that can be reliably detected and amplified by this method. A lower LOD using PCR method reduces the frequency of false negative results.

After each thermal cycle, the number of DNA double strands doubles under optimal conditions. More thermal cycling does not typically result in greater amplification. There are numerous explanations for this. The degradation of PCR components, specifically polymerase, and the decrease in concentration of essential materials in PCR solution are among the most important factors. In addition, a reduction in primer binding or an increase in products that do not match the target results in amplification saturation. The optimal number of thermal cycles should therefore not exceed 30 cycles. Obviously, this number is not fixed and can be reduced based on the PCR technique’s design.

Microfluidics is one of the main paths towards faster, less expensive, POC PCR systems with a smaller footprint as it allows for the miniaturization of fluidic pathways and PCR reactor size while increasing the reactor’s surface-to-volume ratio [8,9]. It also reduces the amount of reagents used and lowers the sample volume required [10,11]. In contrast to conventional tube-based PCR, where 0.2 mL or 0.5 mL tubes are used, the volume of microfluidic PCR reactors might be as low as 100 nL. Because of the much-reduced heat capacity in the latter reactors, ultra-high ramping rates (e.g., 100 °C/s) can be attained [12]. However, a low sample volume also has some adverse effects. One of the drawbacks is the risk of false negative results. Moreover, a low amount of DNA templates in the initial PCR mix leads to a direct reduction in sensitivity [13].

The overall importance of POC PCR for human health came into sharper focus during the COVID-19 pandemic, where PCR became the routine and gold standard testing method for detecting COVID-19. However, implementing PCR in POC settings remains challenging because of its stringent requirements for a low LOD, multiplexing, accuracy, selectivity, sample-to-answer time of 10 min or less, robustness, and cost.

Based on those POC requirements we introduce three parameters to guide the design towards the next generation of RT-qPCR reactors: the overall sample-to-answer time (t); lambda (λ), a measure of the degree to which stochastic effects interfere with obtaining analytical results, thereby setting the minimum number of copies required per unit volume; and gamma (γ), the ratio of thermal cycling time (in min) to the heat cycled sample volume (in mL), which describes the trade-off between the speed of the PCR system and the desired level of sensitivity. It can be interpreted as system efficiency; the most efficient system heat cycles the largest amount of sample in the smallest amount of time. Subsequently, we carry out a numerical simulation and parametric study of a ramping PCR and finally suggest approaches to achieve the PCR reactor for RT-qPCR of the future.

## 2. Design Parameters for POC RT-qPCR

### 2.1. Sample to Answer Time (t)

**Figure 2:**
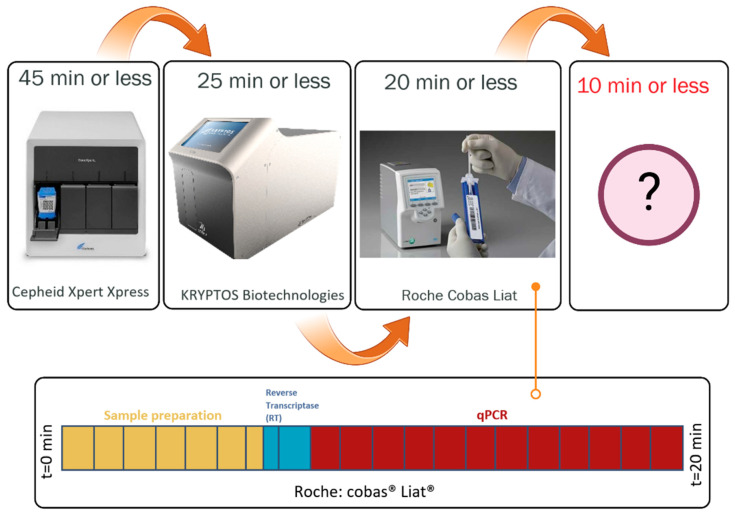
Some more recent RT-qPCR systems that were all developed to tackle COVID-19 and are getting closer to a 10 min sample to answer the goal. The time utilization bar for Roche’s cobas^®^ Liat^®^ shows that from the 20 min sample to answer time, 6.5 min is used for sample preparation (with capture beads, washes, and elution), 1.5 min is for RT, and 12 min or less is for cycling [22].

The first design goal is the overall sample to answer time (t), which we are setting at 10 min or less. This demand is based on the consideration of a desirable sample-to-answer time from a clinical point of view. An investigation into the amount of time doctors claim to spend with a patient reveals that statistically, 13 to 16 min is the most frequently reported estimate. Although it may appear that the amount of time spent with a patient is decreasing by the year, the survey results do not support this and indicate that 13–16 min have remained the prevalent quoted time since 2016 [14,15]. In this context, to cater to more patients, doctors prefer diagnostic equipment and methods with the capacity for a sample-to-answer time of, ideally a few minutes. The recognition of the need for rapid POC-PCR-based diagnostics was further amplified by the COVID-19 pandemic. To prevent the spread of viruses like COVID-19 around the world and to facilitate timely care for patients during a pandemic, rapid screening and diagnosis of viral infections is of vital importance [16,17,18,19]. In RT-qPCR, target sequences of complementary DNAs from viral RNAs are amplified and quantified using real-time reverse transcription quantitative polymerase chain reaction, a technology with a very low LOD and good selectivity [20,21]. A typical RT-qPCR process takes around one hour compared to a 10 min goal. Included in the 1 h sample-to-answer time are sample preparation, reverse transcriptase (RT), thermal cycling, and detection. In Figure 2, we show some more recent RT-qPCR systems that were all developed to tackle COVID-19 and are getting closer to a 10 min sample-to-answer goal. To illustrate how in more recent instruments the sample-to-answer time is divided over sample preparation time, RT, and PCR cycling time, we have put in a time utilization bar for Roche’s cobas^®^ Liat^®^ (Basel, Switzerland) [22]. From the 20 min sample to answer time, 6.5 min is used for sample preparation (with capture beads, washes, and elution), 1.5 min for RT, and 12 min or less for thermal cycling. The large volume (80 µL) ensures good sensitivity.

### 2.2. Limit of Detection–Minimal Number of Copies per Volume (λ)

The second goal is a low enough LOD for the test at hand. In the case of COVID-19, the very best equipment affords an LOD of 100 copies/mL [23]. We are setting our design goal here somewhat arbitrarily at 500 copies/mL. This goal puts an automatic requirement on the amount of samples needed. To understand this, we introduce a parameter called lambda (λ), a measure of the degree to which stochastic effects interfere with obtaining analytical results, thereby setting the minimum number of copies required per unit volume.

**Figure 3:**
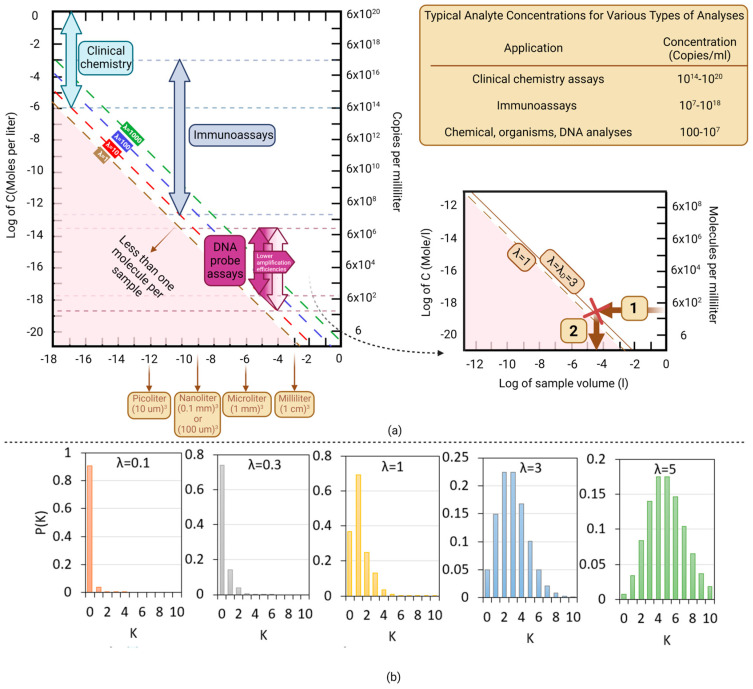
Scaling of concentrations and sample volumes; (**a**) typical chemical and biological concentrations available for a few kinds of analysis. In general sample quantities in the picoliter to nanoliter range are sufficient for assaying the biological molecules involved in clinical chemistry assays (between 10^14^ and 10^20^ copies/mL) and immunoassays (between 10^7^ and 10^18^ copies/mL). A minimum of 100 µL is required for accurate DNA assays (from 10^7^ copies/mL to less than 100 copies/mL). (**b**) The probability that a given partition contains exactly k molecules for different values of λ.

The sample volume required for PCR is primarily dictated by the concentration of the analyte being measured. If a sample input volume with m copies is split/multiplexed over several chambers (n), one must first determine if the statistical distribution of the target DNA molecules across those chambers is a normal distribution or not. When the copy number is very large, the distribution over the n chambers has a statistical variance between samples that is so small that it can be disregarded (m >> n). In such a case, one speaks about aliquoting, and this is the regime for qPCR. In digital PCR (dPCR), the number of targets in the sample (m) is frequently less than or on par with the number of partitions (n). As a result, the number of molecules per partition varies significantly from partition to partition, and one refers to partitioning instead of aliquoting. The average number of targets per partition (λ) is dependent on the sample concentration (C_i_) and the number of partitions (n), or:

(1) $$\lambda =\frac{m}{n}=C_{i}V$$

where V is the total sample volume. Hence, the volume of sample required to detect a particular analyte concentration in a miniaturized system can be calculated from:

(2) $$V_{d}=\frac{V}{n}=\frac{m}{{\eta nN}_{A}C_{i}\;}=\frac{\lambda }{{\eta N}_{A}C_{i}}$$

where $V_{d}$ is the volume of each chamber/partition, N_A_ is Avogadro’s number (6.02 × 10^23^ mol^−1^), C_i_ is the concentration of targeted analyte (mol/L), and η is the sensor efficiency, i.e., the number of photons detected per DNA copy with a value between 0 and 1. When an enzyme is used as a label, an avalanche of photons can be produced per binding event, and this can be captured as η >> 1. During DNA amplification, the initial concentration C_i_ increases as $C_{i}E^{C_{t}}$, where E is the amplification efficiency, and C_t_ is the cycle number. A typical amplification range of 7 to 9 orders of magnitude is shown in Figure 3a.

Today, dPCR is perhaps not ready for POC applications [24]. Nonetheless, it is important that one recognizes when to safely apply a normal statistical distribution of copy numbers for qPCR versus a Poisson distribution for copy numbers in dPCR. The relationship between the concentration and the volume of the chamber/partition is shown in Figure 3a. In the domain below λ=1, there is the probability of chambers/volumes/samples with less than 1 molecule (lowest dashed line). This is the area where the Poisson distribution of particles prevails, and one can use dPCR for absolute DNA quantitation. The other dashed lines apply for larger number of copies per partition (λ=10, 100, and 1000). To be more precise about what value to use for λ for the demarcation of the qPCR domain, we delve a bit deeper into normal and Poisson particle distributions.

When there are n partitions and the partitioning technique is fully random, then the chance of finding the target in a selected partition is 1/n. If this partitioning technique is repeated m times, once for each target molecule, the probability p(k), which gives us the likelihood of k successes or the probability that the given partition contains exactly k molecules is a binomial distribution calculated by multiplying the probability of success raised to the power of the number of successes, and the probability of failure raised to the power of the difference between the number of successes and the number of trials, or

(3) $$p\left(k\right)=\left(\begin{array}{c} m\\ k \end{array}\right)\;{\left(\frac{1}{n}\right)}^{k}{\left(1-\frac{1}{n}\right)}^{m-k}=\left(\begin{array}{c} m\\ k \end{array}\right){\left(\frac{\lambda }{m}\right)}^{k}\;{\left(1-\frac{\lambda }{m}\right)}^{m-k}$$

with $\left(\begin{array}{c} m\\ k \end{array}\right)=\frac{m!}{K!\left(m-k\right)!}\;$ as the bimodal coefficient. Digital assays often include numerous partitions, and with a large n value, the Poisson distribution may be used to approximate the binomial distribution [25]:

(4) $$p\left(k\right)=\frac{\lambda e^{-\lambda }}{k!}$$

In Figure 3b, we plot the probability p(k) for different values of λ [26]. The specific threshold at which to switch from a Poisson to a normal distribution depends on the level of precision required for the analysis. Some statisticians may use a threshold of λ > 5, while others may use a threshold of λ > 10. In practice, to estimate the number of particles needed to achieve the desired level of precision, one may calculate the standard deviation of the measurements and use this value to determine the required sample size.

If one is only interested in a yes or no answer or a semiquantitative determination, the number of molecules per partition necessary for detection (λ_D_ in the inset for Figure 3a) may be chosen at a less stringent λ_D_ = 3. At the intersection of the λ_D_ line and the analyte concentration or copies per mL accessible for analysis, the required sample amount for analysis is determined.

On the other hand, if we want to be quantitative at 500 copies/mL with high precision (λ > 10), one deduces the required sample input volume as follows. First, establish the sample volume that your system can cycle fast enough (see heating efficiency γ goal below), say it is 20 µL and we want to multiplex 5 times, then we need 50 copies, which we can obtain with an input volume of 100 µL. The size of a PCR reactor or the number of available nucleic acid templates has thus a direct effect on sensitivity.

### 2.3. System Thermal Efficiency γ

The total time for any PCR system is the time involved in heating/cooling the reaction chamber until amplification quantitation is possible. As a starting off point, we consider extreme PCR, with a total cycling time < 1 min [27,28]. In extreme PCR, the system is arranged such that the amplification reaction time itself (τ_DNA_) is the slowest step of the whole process. Typically, the slowest step is the extension phase, requiring approximately 1 s or more for a sequence length of 100 bps [29]. Because elongation is the rate-limiting step, a longer amplicon takes a proportionally longer time to amplify. To make τ_DNA_ the slowest step, the reaction volume is limited to 1–5 μL or less, resulting in a high surface-to-volume ratio, which speeds up the heating/cooling process. It is commonly observed that by increasing the concentrations above the suggested ranges, the amplification efficiency increases. However, this usually leads to a lowering of the sensitivity [5]. To illustrate the latter point, Wittwer et al. increased the PCR reagent concentrations, including DNA polymerase and primers, by 15–20-fold above that of a standard PCR mix in order to achieve a PCR time of 1 min (35 cycles) [30]. These remarkable results were obtained by repeatedly transferring a thin capillary from a cold-water bath to a hot-water bath. The small sample size and the cost of the reagents are major impediments to such a method’s commercialization.

According to Figure 3a, when λ decreases (the number of DNA strands per unit volume decreases), a smaller number of DNA strands are required, as well as a proportionally smaller quantity of primers, master mix, etc. This decreases experiment costs. To achieve a lower LOD and reduce PCR reagent consumption, higher volumes are required, but this delays cycling, necessitating a compromise between speed and LOD. Dong et al. [31] introduced the parameter gamma (γ), which aids in quantifying this compromise.

As an example, consider a case with a stringent λ ≥ 10 (to achieve good precision) with a target LOD of 500 copies/mL and multiplexed over 5 PCR chambers (with 3 colors per chamber. This means that 15 different assays can be carried out simultaneously). With a PCR system that features a thermal efficiency γ of 0.4-based on a 20 µL volume with a total cycling time of 8 min (enough to reach a steady state each cycle)-we need at least 50 copies in a distribution chamber of 100 µL, to meet a 500 copies/mL LOD goal. If we aim for 100 copies per mL, as the LOD of the best-in-class assays feature an LOD of ~100 copies of viral RNA per milliliter of transport media [23], and retain the same precision (λ ≥ 10), we need to heat cycle a 100 µL chamber in less than 8 min (γ of 0.08). With the goal of PCR volumes > 20 µL and total cycling times < 8 min, stringent requirements are imposed on heating/cooling rates (power, ramping or shunting, contact or radiative), fluidics (stationary or flow, mixing efficiency) the reactor geometry, walls’ thickness and the thermal conductivity and diffusivity of the materials used in the construction of the PCR reactor.

One cannot discuss sample volume without discussing DNA inhibitors as they affect the amount of sample volume needed for quantitative analysis. Inhibitors may interfere with the DNA amplification process, e.g., by increasing, τ_DNA_ or reducing the efficiency of DNA extraction and purification protocols. As a result, larger sample volumes may be needed to obtain enough DNA copies. Moreover, to cope with the PCR inhibitors, one often dilutes the sample, which may dilute out the PCR inhibitor. It is important then to strike a balance between diluting the sample enough to reduce inhibitor concentration and maintaining sufficient DNA concentration for the application at hand. 

## 3. Numerical Simulation

**Figure 4:**
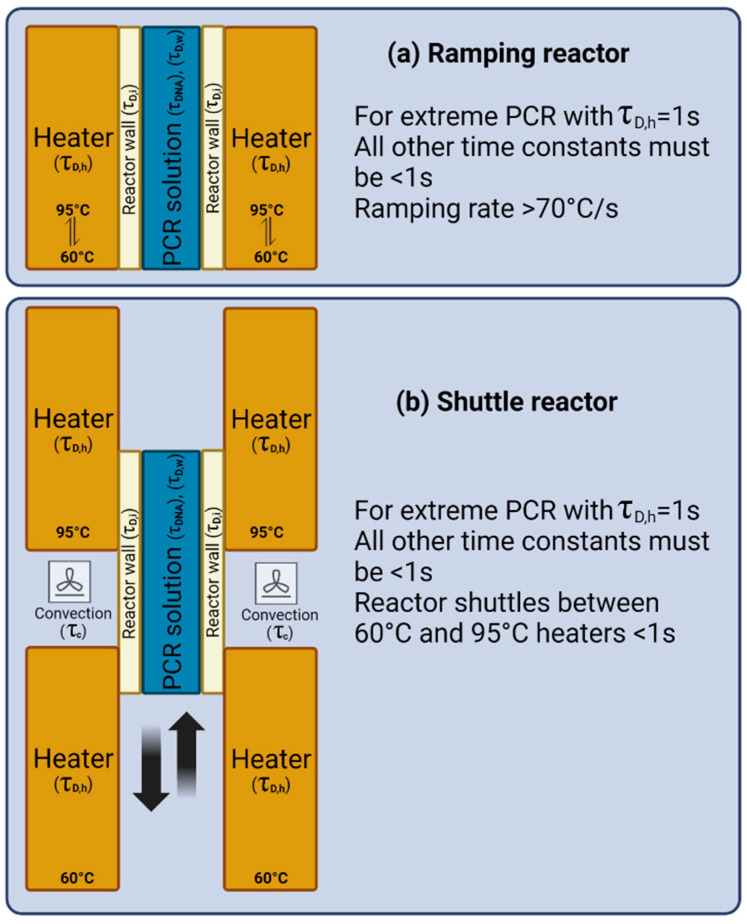
Two typical PCR reactor configurations. (**a**) A ramping PCR system, where the temperature of both heaters on both sides of the reactor is ramped from 60 to 95 °C. (**b**) A shuttling PCR, where the reactor is shuttled from one temperature zone to another briefly exposing the reactor walls to air in between. τ_DNA_ is a time associated with the slowest reaction step in the amplification of DNA. τ_c_ is the time constant for the convection of air over the PCR reactor, $\tau_{D,w}$ is the heat diffusion time for water, $\tau_{D,i}\;$ is the heat diffusion time for the insulating reactor wall, and $\tau_{D,h}$ is the heat diffusion time for the heater.

We now set up a numerical simulation to help develop the next generation of realistic PCR reactors for POC with a sample-to-answer time of 10 min or less, a lower LOD, less expensive systems, and fewer reagents. In Figure 4, we illustrate two typical reactor configurations: in Figure 4a shows a ramping PCR system, where the temperature of heaters on both sides of the reactor is ramped from 60 to 95 °C and back down; and in Figure 4b, a shuttling PCR is shown, in which the reactor is shuttled from one temperature zone to another, briefly exposing the reactor walls to air in between.

We will calculate the time it takes for a PCR reactor to reach temperature uniformity to within 1 to 2 °C, ensuring that samples are subjected to the same temperature so that denaturation, annealing, and extension steps occur optimally. If the temperature is not uniform across the samples, some may experience sub-optimal conditions, leading to incomplete denaturation, inefficient annealing of primers, or incomplete extension of the DNA. This can result in lower yields or inaccurate results. Moreover, temperature gradients within each sample lead to variations in the efficiency of the PCR reaction. These variations can cause different amplification rates for different areas in the reactor, leading to inaccurate quantification and representation of the initial DNA sample.

To calculate the time duration of each contributing τ from Figure 4 to the overall PCR time, we need to introduce some critical material properties and some basic equations. In Figure 4, τ_DNA_ is a time associated with the slowest reaction step in the amplification of DNA. As we saw earlier, in extreme PCR, with a total cycling time < 1 min, the system is arranged such that τ_DNA_ is the slowest step of the whole process. Typically, that is the extension, requiring approximately 1 s or more for a sequence length of 100 bps. In other words, for the reaction rate limitation to be observed, all the other steps in the process must be shorter than 1 s. In two-step PCR, one heat cycles is typically between 60 °C and 95 °C, so we need temperature ramp rates of 70 °C/s or more for heating and cooling to achieve the <1 s target, as beyond this ramp rate, the reaction rate controls. Similarly, for shuttling (close, heat, open, move, close, heat) we need this time also to be less than 1 s.

Next, we need to address the heat flow at the interfaces between heater elements and the PCR reactors. Two solid surfaces in contact transfer heat in two modes: conduction at solid-to-solid contacts, which can be very effective and conduction through the air-filled gaps, which, due to air’s low thermal conductivity, is very poor. To treat the thermal contact resistance at such a contact interface, we write out Newton’s cooling law for q, which is the rate of heat that transfers out of the body. Similarly to a convection heat transfer coefficient h, an interfacial conductance h_c_, with the same units of W/m^2^·K, is introduced:

(5) $$q=-h_{c}\times A\times ∆T$$

The thermal contact resistance R_t_ = 1/(A·h_c_) depends on the surface finish of the contacting faces, the material of each face, the pressure applied to the contact area, and the air in the gaps between the two contacting faces.

Figure 4b has the additional time τ_c_ associated with the convection of air over the PCR reactor:

(6) q=−h×A×∆T

where h is the convection heat transfer coefficient in the air with units of W/m^2^·K, A is the area of the reactor, and ΔT is the difference between the reactor temperature and the temperature of the air in contact with the reactor. For a reactor at a surface temperature of 95 °C in still air, the convective heat transfer coefficient by natural convection is around 5–10 W/(m^2^·K), and in the case of forced air flow, the convective heat transfer coefficient can be around 50–100 W/(m^2^·K) (with a flow velocity of 2 m/s). For the current analysis, given that the shuttling time for the reactor is <0.5 s and that there is very little time for convection cooling, we will ignore this term.

### 3.1. Governing Equations and Boundary Conditions

For a time- and space-dependent temperature variation, we must solve the heat diffusion Equation (7).

(7) $$\frac{1}{\alpha }\frac{\partial T}{\partial t}=\frac{\partial^{2}T}{\partial x^{2}}+\frac{\partial^{2}T}{\partial y^{2}}+\frac{\partial^{2}T}{\partial z^{2}}$$

where T is the temperature, t is the time, and α (m^2^/s) is the thermal diffusivity. To establish steady-state heat transfer, heat must diffuse first across a material, and in the case of fast PCR, it is essential to have a quick thermal response of a bulk material when changing its surface temperature. The swiftness of this response depends on the diffusivity α of the material, i.e., the ratio of thermal conductivity, k (W/m·K), to volumetric heat capacity, C_p_ (J/(m^3^·K), and the material density, ρ (kg/m^3^).

(8) $$\alpha =\frac{k}{\rho C_{p}}$$

Diffusivity α measures the capacity of a material to transport thermal energy compared to its ability to store thermal energy. In other words, materials with a larger α react quickly to variations in their thermal environment, whereas materials with a smaller α react sluggishly, taking longer to achieve a new equilibrium state. Thermal diffusivity is more appropriate than a material’s thermal conductivity for calculating the time required to heat a reactor because it considers both the material’s thermal conductivity and its specific heat capacity. In the case of gene amplification, the bulk materials include both the PCR solution, the solid walls of the chamber, and the heaters. To measure the time, it takes to diffuse over a diffusion distance of d, one calculates heat diffusion time $\tau_{D}$:

(9) $$\tau_{D}=\frac{d^{2}}{\alpha }$$

For each material in the reactor, we have such a heat diffusion time, $\tau_{D,w}$ for water, $\tau_{D,i}$ for the insulating reactor wall, and $\tau_{D,h}$ for the heater. In Table 1, we list the thermal characteristics of common materials used in PCR devices. For our numerical simulation we used the thermal conductivity, diffusivity, density, and specific heat capacity of polypropylene (PP), one of the most frequently used reactor materials. For each of these materials, it takes a time t > $\tau_{D}$ to reach steady state, i.e., temperature uniformity. For solids, diffusivity is the only available thermal transport mode, for fluids, convection, diffusion, and mixed convection/diffusion are possible. Of course, convection, especially forced convection, introduces additional complexity, such as flow control systems. An elegant solution, avoiding the complexity of a flow system, is one where reciprocation or squeezing of the reaction chamber is employed (see reactors 1 and 4 in Table 2). 

As the boundary conditions, we applied constant temperatures of 95 °C to the outer boundaries of the heaters. As the initial conditions, we applied 95 °C to the heaters and 60 °C to the insulators and water samples.

### 3.2. Geometry and Dimensions

**Figure 5:**
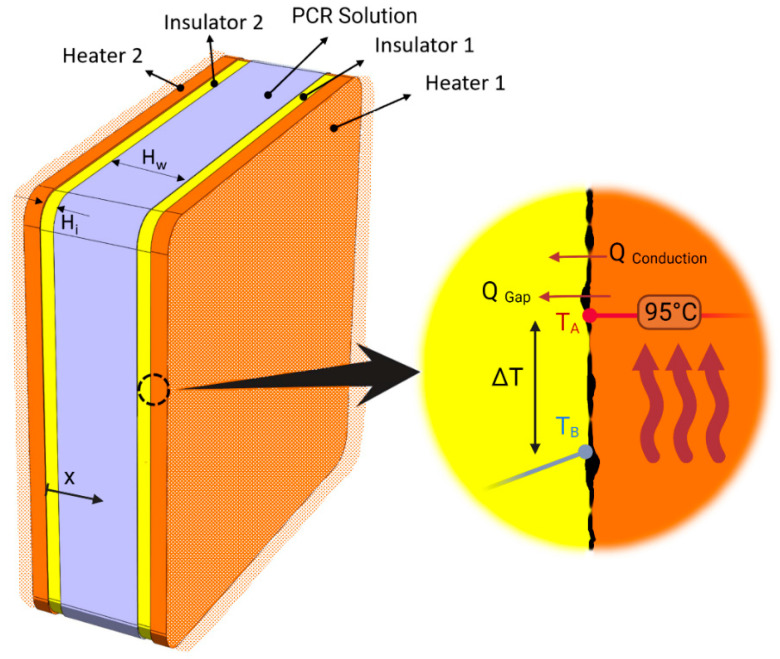
Simulation domains and contact resistance between the heater surface and insulator. Orange layers show a thin layer (thickness = 300 μm) of heater with a constant temperature of 95 °C; yellow layers show the insulator (thickness = 25 µm to 150 µm); and the blue layer shows the PCR solution considered as water (thickness = 100 µm to 500 µm). Only a thin layer (300 µm) of heater very close to the interface has been considered.

We considered a constant cross-sectional area with an insulator (PP) thickness of H_i_ and a chamber height of H_w_. As shown in Figure 5, a contact resistance of R_f_ between the insulator and the heater’s (e.g., copper) surface was considered. Thermal contact resistance depends on the applied pressure of the heater, the nature of the contacting materials, and the roughness of the interface region. For representative values of R_f_, we used the thermal contact resistance vs. pressure as given in [38] for the case of stainless steel on polycarbonate.

### 3.3. Solving Method

All the governing equations were discretized using the finite element method and solved simultaneously. Thus, following the grid independence test, a total of 170,000 structured elements were used to discretize the geometry. The iterative method of GMRES with a convergence criterion of 0.001 was used to obtain solutions [39,40,41].

### 3.4. Results and Discussions

**Figure 6:**
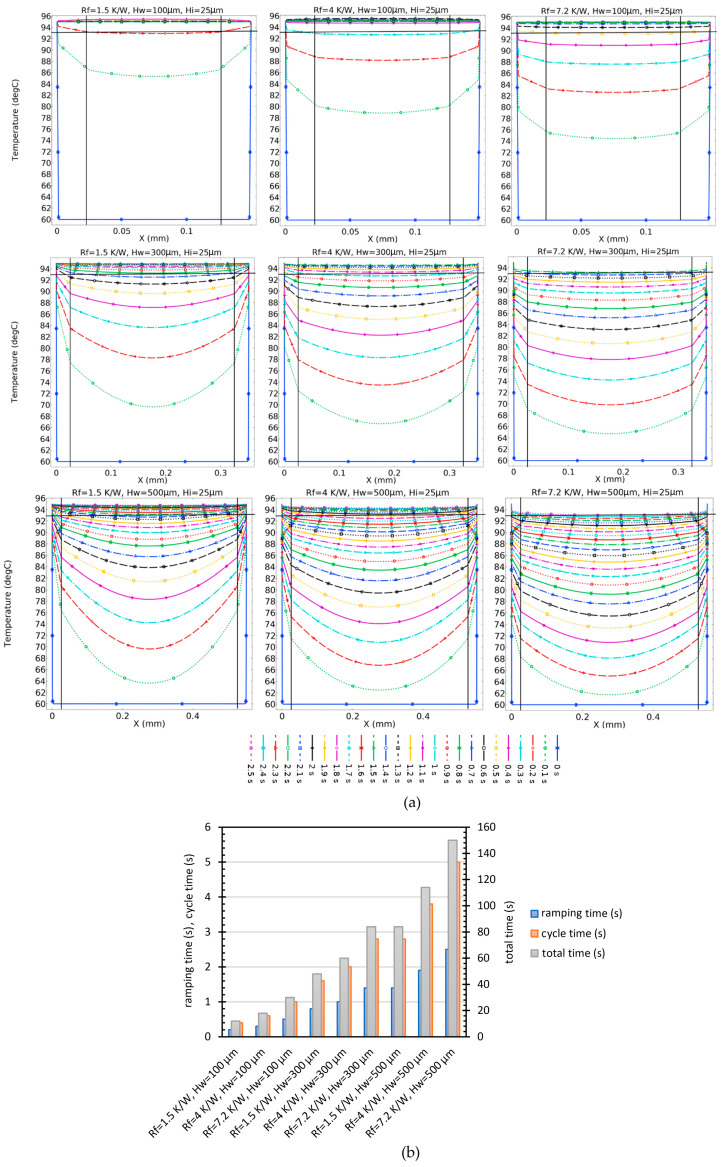
(**a**) Effect of chamber height and contact resistance on temperature distribution over time. The solid vertical black lines mark the insulator-to-solution transition and the horizontal line at 93 °C marks the maximal allowed spread of reactor mix temperature (2 °C). (**b**) Ramping, cycle, and total times of different configurations of contact resistance and water heights.

In Figure 6, we depict the transient temperature distribution along the central line of a PCR chamber with two heaters (one on each side), a 25-µm thick layer of PP in contact with the heaters, and three varying chamber heights. In each case, we look at three different contact resistances: 1.5, 4, and 7.2 K/W. For a low chamber height (H_w_ = 100 µm) and low contact resistance (1.5 K/W), temperature uniformity (within 2 °C) is reached within 0.2 s. With a chamber height of 500 µm and the same thermal resistance temperature, uniformity is reached only after 1.5 s. Comparing a 100 µm and a 500 µm reactors to the same thin 25 µm reactor walls, but with the high thermal contact resistance of 7.2 K/W, results in a time-to-temperature uniformity of about 0.5 s for the 100 µm reactor, but the 500 µm does not even reach that condition after 2.5 s.

**Figure 7:**
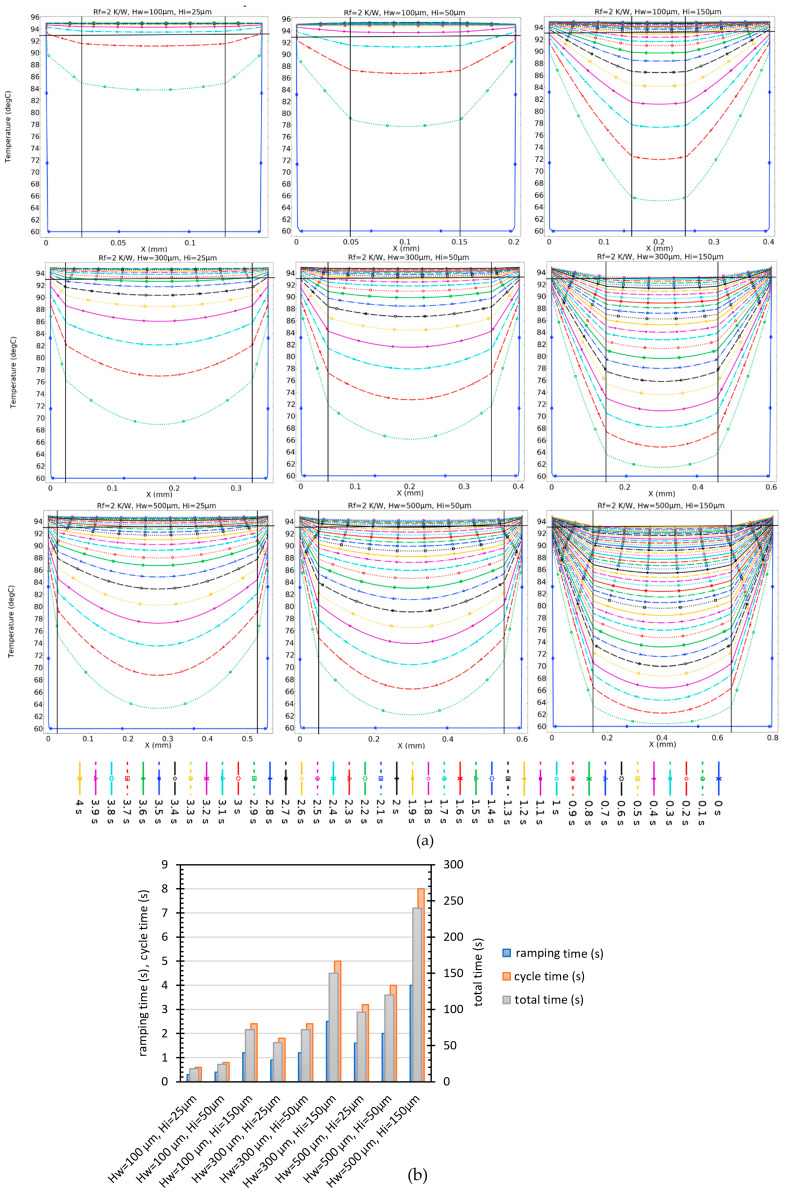
(**a**) Effect of insulator thickness on temperature distribution over time with the constant contact resistance of 2 K/W. The solid vertical black lines mark the insulator-to-solution transition and the horizontal line at 93 °C marks the maximal allowed spread of reactor mix temperature (2 °C). (**b**) Ramping, cycle, and total times of different configurations of insulator thickness and water heights.

In Figure 7, the dramatic effect of the insulator thickness for different PCR solution chamber heights and a contact resistance of 2 K/W is depicted. This figure indicates that uniformity occurs in a ramping time of 0.3 s for a thickness of 25 µm and chamber height of 100 µm. However, by increasing the insulator thickness to 150 µm, the time to equilibration increases to 1.4 s, and with the same insulator thickness, a 500 µm reactor does not even approach uniformity after 4 s.

The simulation results above apply to static PCR chambers with contact heating and no convection in the reactor itself. From the above, we saw that the diffusion time can be defined as $\tau_{D}=\frac{d^{2}}{\alpha }$. This diffusion time, in the absence of convection, is given by $t_{diff}=\frac{d^{2}}{D_{t}}$. If convection aggressively stirs the fluid and rapidly decreases the length scales over which diffusion must occur, then the mixing time is much smaller than $\tau_{D}$. In Figure 6 and Figure 7, this would result in the temperature profiles in the liquid becoming uniform much faster and the time lags caused by the thermal resistance associated with the insulator and the thermal contact resistance becoming more prominent.

## 4. Candidate PCR Reactors in the Market or under Development

With the overall sample to answer time (t); lambda (λ), which locks in the LOD; gamma (γ), the thermal efficiency; and the above simulation results in mind, we made a thorough literature review of PCR reactor designs. In Table 2, we tabulate those PCR reactors that are under development or on the market, and we were able to calculate γ from the data provided. Only results from PCR experiments where total cycling time was below 10 min (except for the Roche’s cobas^®^ Liat^®^ at 12 min) and/or had a volume equal to or above 20 µL were retained. Using these criteria, most reactor designs were rejected (4 accepted, 22 rejected). From the four PCR reactor designs that are based on contact heating that qualify, two are microplate-based and not geared towards POC. The two systems (NextGenPCR^®^ (Goes, The Netherlands) [42] and Cobas^®^ Liat^®^ (Basel, Switzerland) [22]) with the lowest gamma introduce convection by squeezing the reactor chamber, and a relatively low thermal contact resistance can be surmised because of the pressure exerted on the reaction chamber. Introducing convection by squeezing is a much simpler way than relying on flow, which requires a control system. The two static reactors in the table can only achieve better heating efficiencies by reducing the PP film even further (40 µm for Lex Diagnostics (Royston, UK) and 10 µm for Xxpress™ (Duluth, GA, USA) [43]) and by minimizing the heat transfer resistance further. The latter is achieved either by making the heater part disposable (Xxpress™) or employing an efficient heat spreader material (Lex Diagnostics).

Two PCR systems in the research stage deserve some extra attention. The first one, uses thermalized liquids switching to contact the static reactor walls and is made for smart and effective heat coupling, for which a γ of 7.5 min/25 µL = 0.3 was achieved using a 50 µm polypropylene reactor wall [44]. Injections of hot water instead of air increase the heat transfer rate by eight times and have better temperature uniformity in comparison to air thermalization. The two heat transfer fluids (for annealing/elongation and the denaturation temperature) are sequentially transferred into the heat exchangers and then into the thermalization chamber, and their flow is controlled with a pressure controller. This system executed 30 RT-PCR cycles in 7.5 min with regular microliter-sized samples (25 µL) and a 0.03 °C thermalization homogeneity. Second is the reactor by Yi-Quan et al., using a static shuttling reactor, achieved a γ of 9 min/25 µL = 0.3, using a 50 µm polypropylene reactor wall and using pressure on the heater to heat spreader film and pressure and silicon grease between the heat spreader films and the reactor chamber [45]. Both efforts demonstrate that the best way to improve a static thermal-diffusion-only reactor is by minimizing the thermal contact resistance.

## 5. Conclusions

In this paper, we presented essential design criteria for RT-qPCR devices. We proposed three essential parameters to guide the design of the next generation of PCR reactors: the overall sample-to-answer time (t), lambda (λ, which determines the minimum number of copies required per reactor volume), and gamma (γ, the thermal efficiency of the system). In addition, we have developed a numerical model that demonstrates the effect and significance of contact resistance between the heat source and the PCR solution-containing reservoir’s walls, as well as wall thickness and sample volume (i.e., reservoir height), on the rate and uniformity of heat diffusion. At a constant wall thickness and contact resistance, we demonstrated that increasing the sample volume by a factor of five increases the time required to reach a uniform temperature by a factor of up to seven. Increasing the wall thickness by a factor of 6 increases the time by a factor of 2.5 up to a factor of 4. The duration is increased by a factor of 1.7 to 2.5 by increasing the contact resistance between the heater and wall. Finally, we compared our criteria to devices that are commercially available.

## Figures and Tables

**Table 1:** Thermal characteristics of common materials used in PCR.

	Material	Thermal Conductivity (W/m·K)	Density (kg/m^3^)	Specific Thermal Capacity (kJ/kg K)	Thermal Diffusivity (m^2^/s)	Ref.
Solids	PMMA	0.21	1202.9	1.46	1.19 × 10^−7^	[32]
	PDMS	0.15	970	1.46	1.06 × 10^−7^	[33]
	PC	0.2	1226.7	1.22	1.34 × 10^−7^	[32]
	COC 8007	Between 0.12 and 0.15	1020	1.2	1.17 × 10^−7^	[34]
	PP	0.22	900	1.330 to 2.400	0.961 × 10^−7^	[35]
	Silicon	148 (3.2 to 150)	2330	0.712	9 × 10^−5^	[36,37]
	Teflon	0.35	2200	0.97	1.64 × 10^−7^	[36]
	Glass	1.4	2500	0.75	7.46 × 10^−7^	[36]
Liquid	Water	0.613	997	4.17	1.47 × 10^−7^	[36]
	Oil	0.131	837	1.67	7.85 × 10^−8^	[36]

**Table 2:** Selected commercialized or under development PCR devices.

Type of System	Device/Company	Time to Result	Characteristics	Thermal Contact Resistance	γ = Time/Volume
Plate system, not an integrated system, up to 96 wells	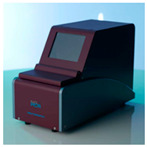 NextGenPCR^®^ MBS Molecular Biology Systems (Goes, The Netherlands)	2 min without sample preparation	- Shuttling (3 temperature zones) < 1 s - Static reactors - Heater on both sides - τ_DNA_ observed? Yes - Convection: squeezing - PP thickness < 50 µm	Low (squeezing pressure)	2 min/20 µL = 0.1
Integrated POC system	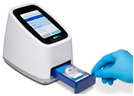 Lex Diagnostics (Melbourn, UK)	Sample to answer of 5 min and thermal cycling time of less than 3 min	- Ramping (>75 °C/s) - Static reactor - Heater on one side - No convection - PP thickness = 40 µm	Very low (engineered permanent contact)	3 min/5 µL = 0.6 With heater on both sides: 3 min/10 µL = 0.3
Plate system, not an integrated system, up to 96 wells	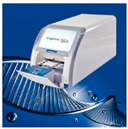 XXPress BJS Technologies (Duluth, GA, USA)	10 min without sample preparation	- Ramping (>10 °C/s) - Static reactor - Heater on one side - τ_DNA_ observed? - No convection - PP thickness = 10 µm	Lowest (engineered permanent contact)	10 min/40 µL = 0.4
Integrated POC system	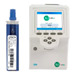 Roche’s Cobas Liat (Basel, Switzerland)	20 min with sample preparation, 12 min for PCR	- Shuttling - Static reactor - Heater on one side - τ_DNA_ observed? - Convection by heat stamps - PP thickness = 65 µm	Low (squeezing pressure)	12 min/80 µL = 0.15

## Data Availability

The data presented in this study are available on request from the corresponding author.
